# Larger Sizes Matter More! Applying the Matteo Mathematics Method for Endovascular Aortic Bifurcation Reconstruction to Large Venous Vascular Repair

**DOI:** 10.7759/cureus.3537

**Published:** 2018-11-02

**Authors:** Jerry Matteo, Preston Hood, Paul C Hulsberg, Erik Eadie, Erik Soule, Michael Shabandi, Taylor S Harmon

**Affiliations:** 1 Interventional Radiology, University of Florida College of Medicine, Jacksonville, USA; 2 Interventional Radiology, The University of Texas Medical Branch, Galveston, USA

**Keywords:** endovascular repair, kissing stents, native vessel, matteo mathematics method, inferior vena, aorta, iliac graft, endoleak, trauma, atherosclerosis

## Abstract

Endovascular aortobifemoral bypass repair with aortic bifurcation reconstruction is a well-established option with mortality benefits compared to conventional surgical management. The same theory, formulas, and techniques can be applied to the central venous system as long as there are commercially available devices. Using mathematically derived criteria for optimal stent size selection, endovascular aortic bifurcation reconstruction with kissing stents was extrapolated to the inferior vena cava (IVC). This report describes a traumatic case of IVC injury that was successfully repaired using the standard aortic grafts while adhering to the guidelines for proper stent size selection.

## Introduction

Arteriosclerotic vascular disease (ASVD) progression varies between patients and is treated based on the severity of manifestations. Historically, in the most severe cases, revascularization of the lower extremities secondary to aortoiliac ASVD involves major intra-abdominal surgery with aortobifemoral bypass grafting. Surgical management entails a perioperative mortality rate of as high as five percent [1]. Endovascular aortobifemoral bypass repair with aortic bifurcation reconstruction has recently become a conventional interventional procedure, offered to patients who are not appropriate surgical candidates [2].

In the case of endovascular aortic repair, a subset of patients with ASVD has small caliber vessels, rendering repair of the aorta and iliac arteries incompatible with the available endovascular stent grafts [3]. The physical size burden of these endovascular delivery systems requires a patent vessel lumen diameter of at least 7 mm [4]. To enable endovascular treatment of such patients, a novel percutaneous piecemeal stent graft placement technique was described by Matteo et al. This technique utilized individual placement of the device components to circumvent the size limitations of the available deployment systems. A mathematical method was derived for the selection of an ideal stent graft size to avoid infolding and endoleaks [5].  

This piecemeal endovascular approach was adapted to the central venous system, where the inferior vena cava (IVC) was reconstructed in a similar manner. As the aortoiliac bifurcation is repaired for ASVD with appropriately sized side-by-side stents, the IVC and bifurcating iliac veins can likewise be repaired in the setting of trauma. Traumatic iatrogenic injury of the IVC occurred during placement of a laparoscopy trocar in a 70-year-old male. This was treated with endovascular IVC reconstruction, extrapolating the mathematical method previously derived by Matteo et al. for stent size selection to the venous system.

## Materials and methods

**Figure 1:**
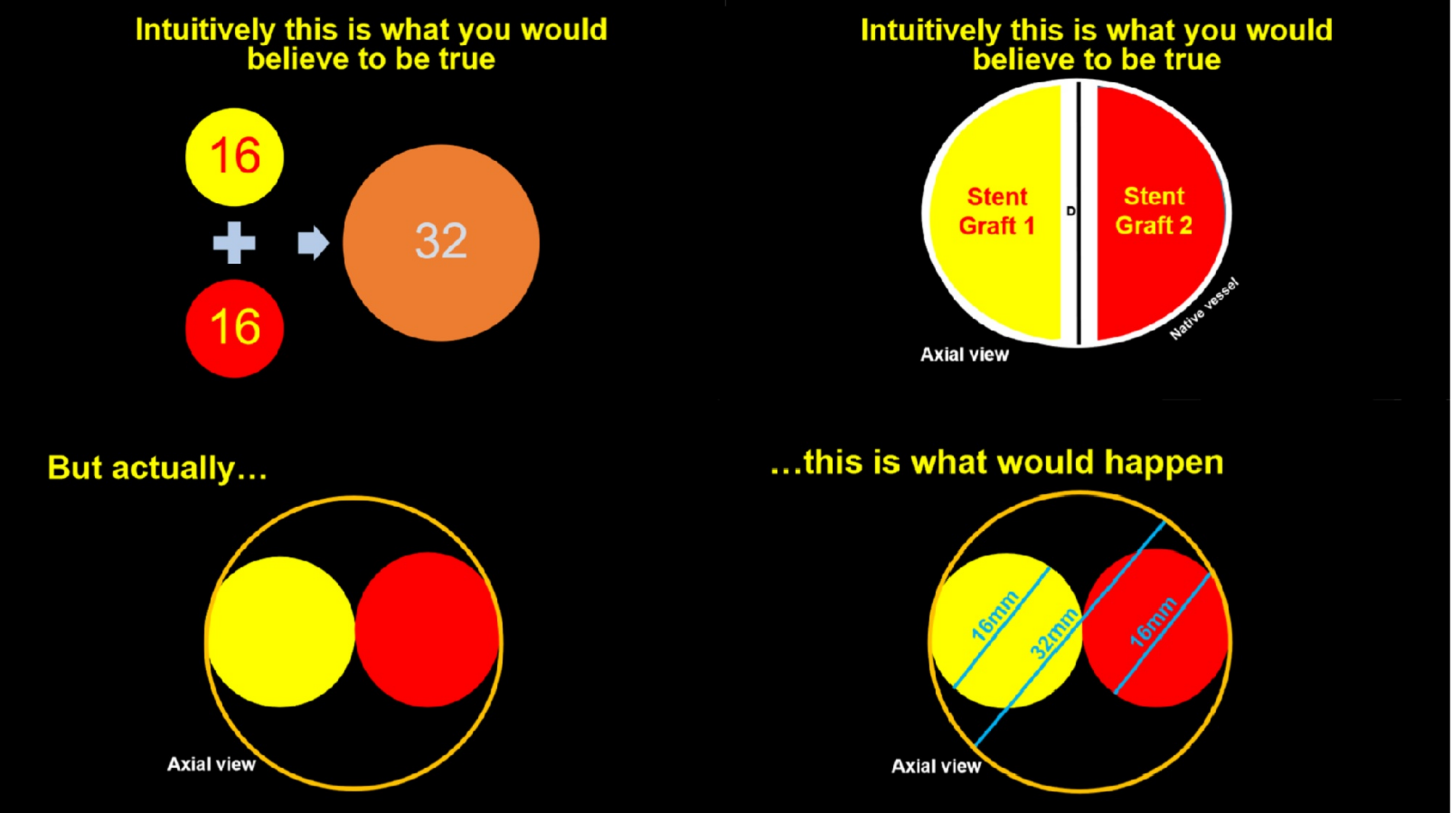
The non-intuitive nature of iliac stent placement Selecting the iliac stent diameter size is paradoxical since calculating the native vessel size (aorta or inferior vena cava) is not as intuitive as adding the diameters of the patient's iliac vessel lumens together. Doing so will lead to the infolding and endoleaking of the bifurcation graft, resulting in repair failure. As shown, iliac stent diameters of 16 mm will not result in a tight seal to accommodate a native vessel (aorta or inferior vena cava) or 32 mm. Instead, though the iliac stents will traverse the diameter of the native vessel (aorta or inferior vena cava) superfluous space will remain between the iliac stents and the native vessel (aorta or inferior vena cava) stent.

The diameter of a native vessel lumen can be accurately obtained using the interface tools on various imaging modalities. In the case of a diseased aortoiliac bifurcation in ASVD, or an IVC that has undergone vessel trauma, the diameter of the native vessel lumen is the first step in a sequence that will determine the "kissing" or side-by-side stent sizes to be deployed in the iliac arteries and veins, respectively. It seems intuitive that the corresponding piecemeal stent diameter of choice used to repair a large vessel bifurcation would be the total diameter of the native vessel (aorta or IVC) divided by two. For example, if the native vessel diameter of the aorta or IVC to be repaired is 32 mm, it is paradoxically incorrect to place two piecemeal stents with a 16-mm diameter in each iliac artery or vein, respectively. In practice, this will lead to an inadequate repair of these vessels, leading to stent graft endoleaks (Figure 1).

In this case, the Matteo mathematics method is used to calculate accurate piecemeal stent sizing:

$$1.05(\pi D_{Native Vessel}) = (2\pi D_{Stent Graft}) - (2 D_{Native Vessel})$$

The Matteo mathematics method states that the circumference of the stent to be placed in the native vessel lumen (aorta or IVC) should be equal to the combined circumferences of the side-by-side stent grafts placed in the iliac arteries or veins, minus two times the diameter of the native vessel (aorta/IVC) wall to account for the redundant material at the interface of the kissing stents. Of note is the five percent stent upsize value, demonstrated by 1.05, which accounts for stent oversizing as suggested by the manufacturer. Using the interface tools, the diameter of the aorta or IVC can be measured and inserted into the above equation to solve for the side-by-side stent diameters necessary to adequately repair an injured or diseased bifurcation.

There are instances where the side-by-side stents are differing calibers based on the corresponding iliac vessel lumens. In this case, the modified Matteo mathematics method must be considered to calculate for the differing kissing stent diameters, which requires the appreciation of the stent redundant material between them. Regardless of the side-by-side stent diameters needed to ensure the vessel bifurcation is adequately repaired without endoleaks or infolding, the redundancy of graft material is a constant value. This should first be solved to determine the different side-by-side stent diameters:

$$1.05(\pi D_{Native Vessel}) = [(\pi D_{Large Stent Graft}) + (\pi D_{Small Stent Graft})] - X_{Total Redundant Material}$$

$$X_{Total Redundant Material} = [(\pi D_{Large Stent Graft}) + (\pi D_{Small Stent Graft})] -1.05(\pi D_{Native Vessel})$$

The stent redundant material solved for accounts for the portion where the two opposing stents contact each other and not the lumen of the receiving vessel. After calculating this value, any stent diameter can be solved. Therefore, if the native vessel (aorta or IVC) diameter is known, the corresponding equal size or unequal size stents can be properly achieved.

The Matteo mathematics method as described can be applied to both arterial and venous systems as mentioned; however, the latter has never been documented. The following case will demonstrate the application of the Matteo mathematics method to the repair of a traumatized IVC, where the iliac stent diameters were calculated to achieve the best fit.

**Figure 2:**
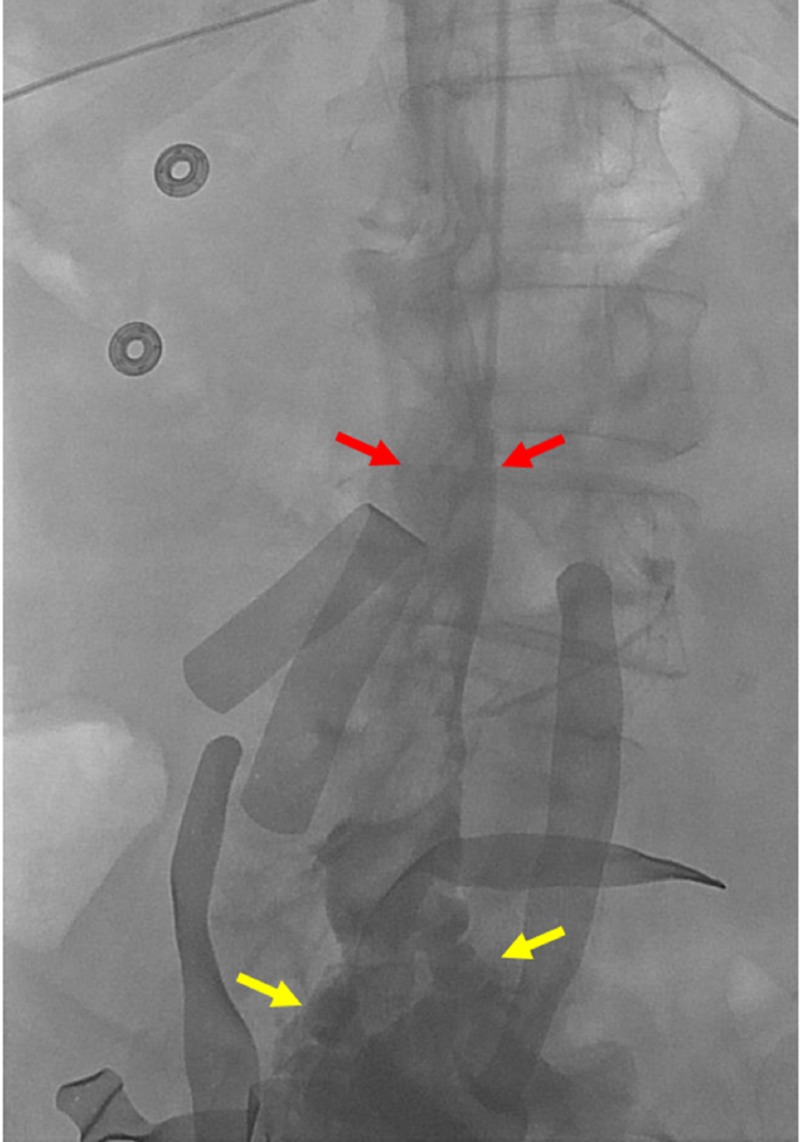
Initial cavogram accessed from the jugular vein Initial cavogram from the transjugular venous sheath demonstrates partial opacification of the inferior vena cava (red arrows), with large amount of extravasation into the pelvis (yellow arrows).

**Figure 3:**
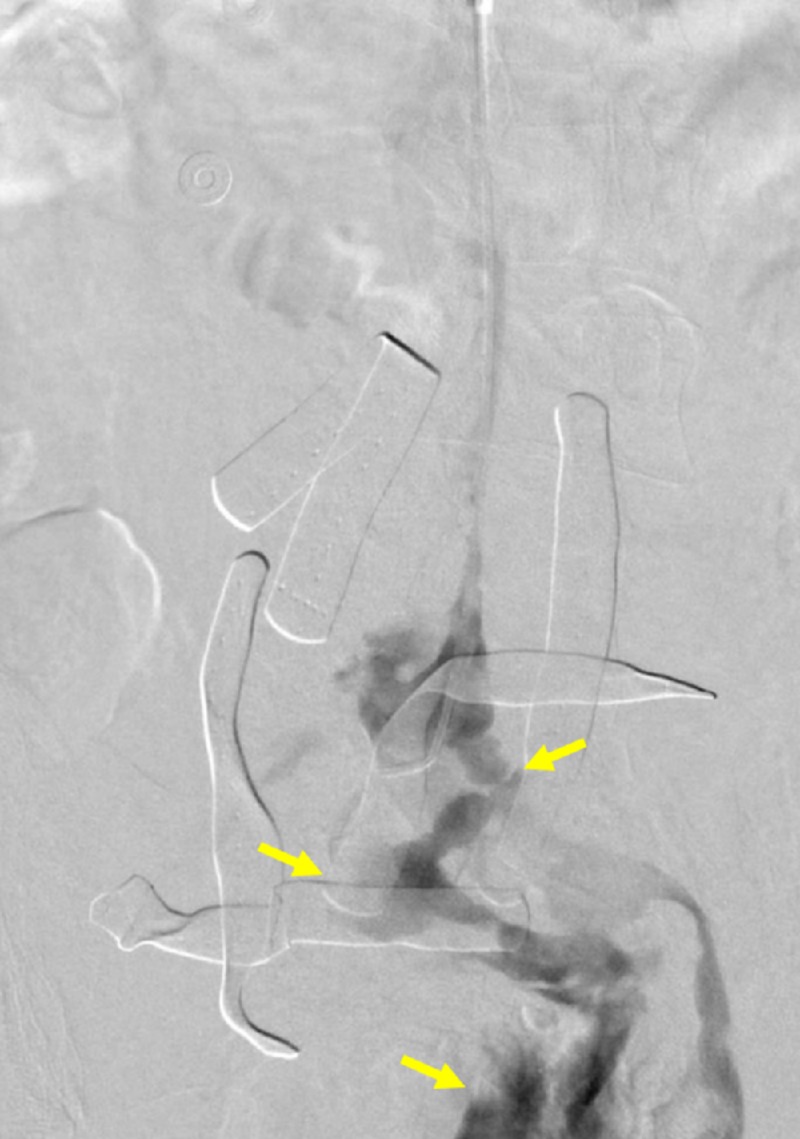
Initial venogram demonstrating reflux and extravasation into the pelvis A venogram performed through a transjugular venous catheter demonstrates partial reflux into the left common iliac vein and large amount of extravasation into the pelvis (yellow arrows).

A 70-year-old male, surgically managed for esophageal cancer, presented for traumatic iatrogenic injury of the aorta and IVC that occurred during the placement of a laparoscopy trocar. The patient became hemodynamically unstable, entered hemorrhagic shock, and was subsequently stabilized. Open repair of the aortic lesion and fasciotomies for compartment syndrome of the bilateral legs were conducted. The IVC injury was not identified during the laparotomy; however, interventional radiology was consulted for the IVC filter placement due to concern for pulmonary embolus (PE). After the right internal jugular vein (IJV) access was obtained, an IVC venogram revealed massive contrast extravasation into the peritoneum and pelvis (Figures 2-3).

At this time, the decision was made to treat the IVC injury by initially gaining access to the bilateral common femoral veins. Five French vascular sheaths were upgraded to 18 French sheaths. Stiff Lunderquist® guidewires (Cook Medical, Bloomington, Indiana) were advanced with the aid of Berenstein catheters, and the sheaths were positioned in the IVC.

**Figure 4:**
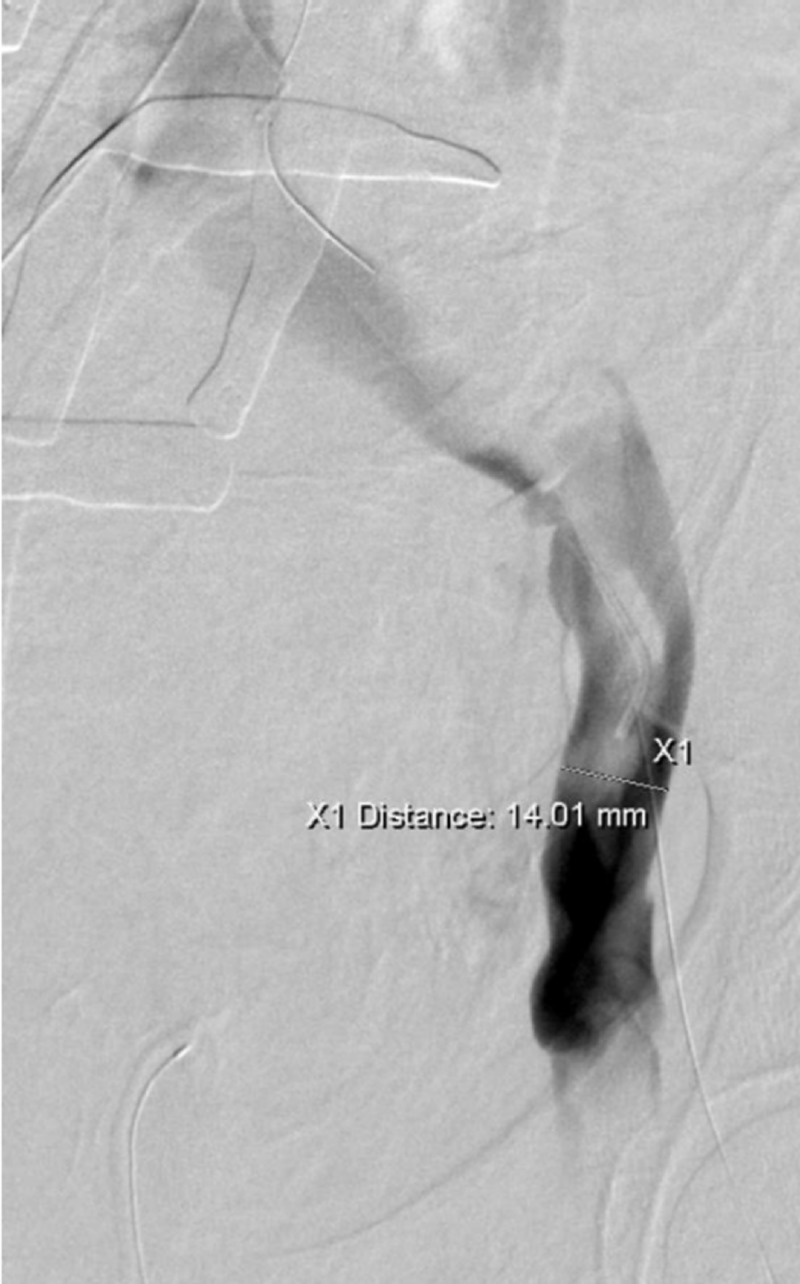
Thrombus formation demonstrated by left external iliac venogram A left external iliac venogram through the right common femoral vein access demonstrates intraluminal filling defects consistent with the thrombus. The iliac vein was measured in preparation for covered stent placement.

The renal veins were then selectively catheterized and positions marked prior to the placement of the IVC stent graft (Figure 4).

**Figure 5:**
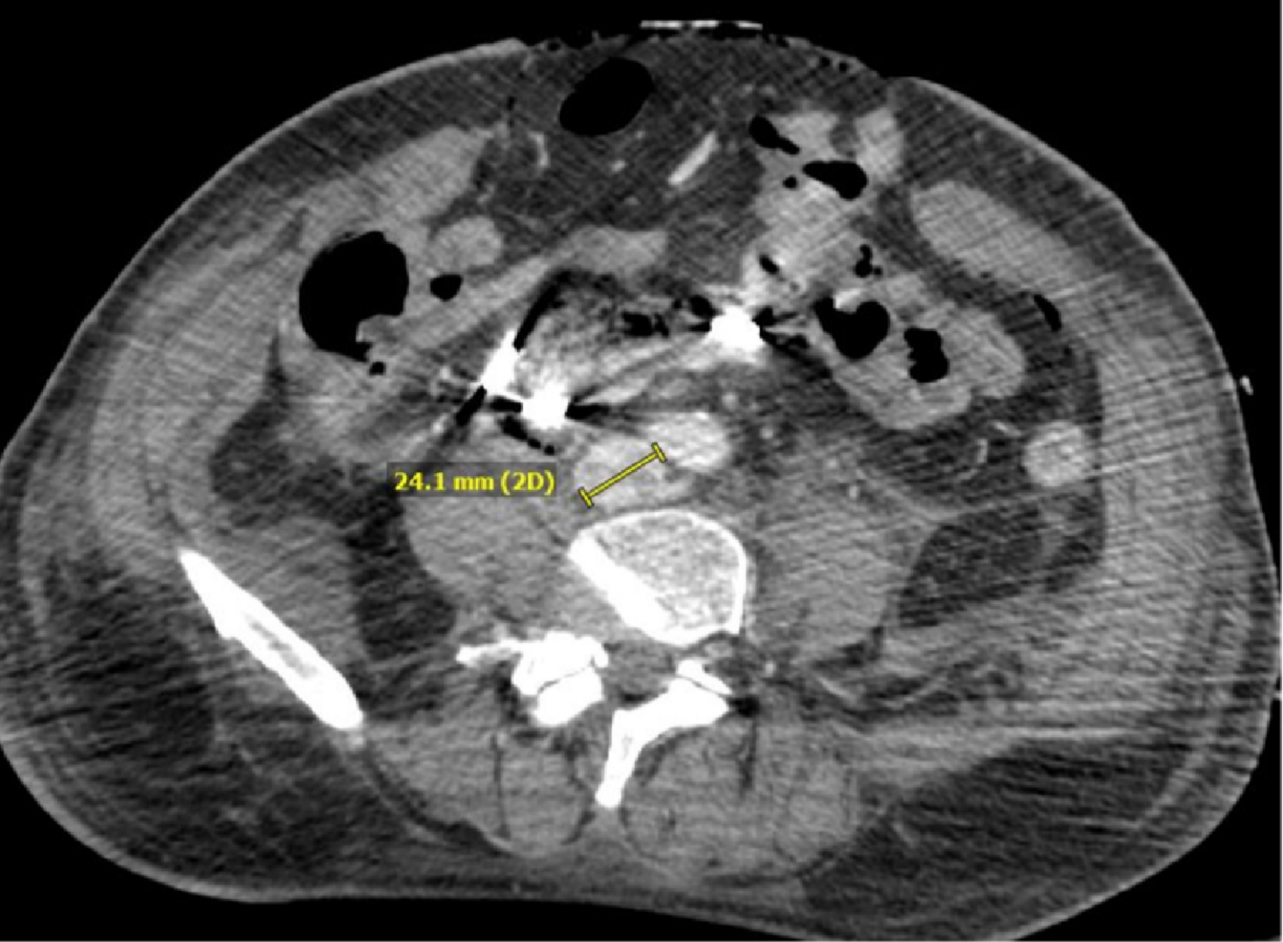
Axial CT of the measured inferior vena cava An axial CT of the patient's measured inferior vena cava is shown, with the diameter being 24.1 mm. This is considered the native vessel diameter and can be used to calculate the side-by-side iliac stent diameters needed for adequate bifurcation repair. CT: computed tomography; IVC: inferior vena cava

After repositioning of the Lunderquist® guidewire, a pigtail catheter was introduced into the IVC, confirming positioning within the central aspect. In preparation for side-by-side iliac stent placement, computed tomography (CT) was utilized to determine that the native vessel diameter of the IVC was 24.1 mm (Figure 5).

**Figure 6:**
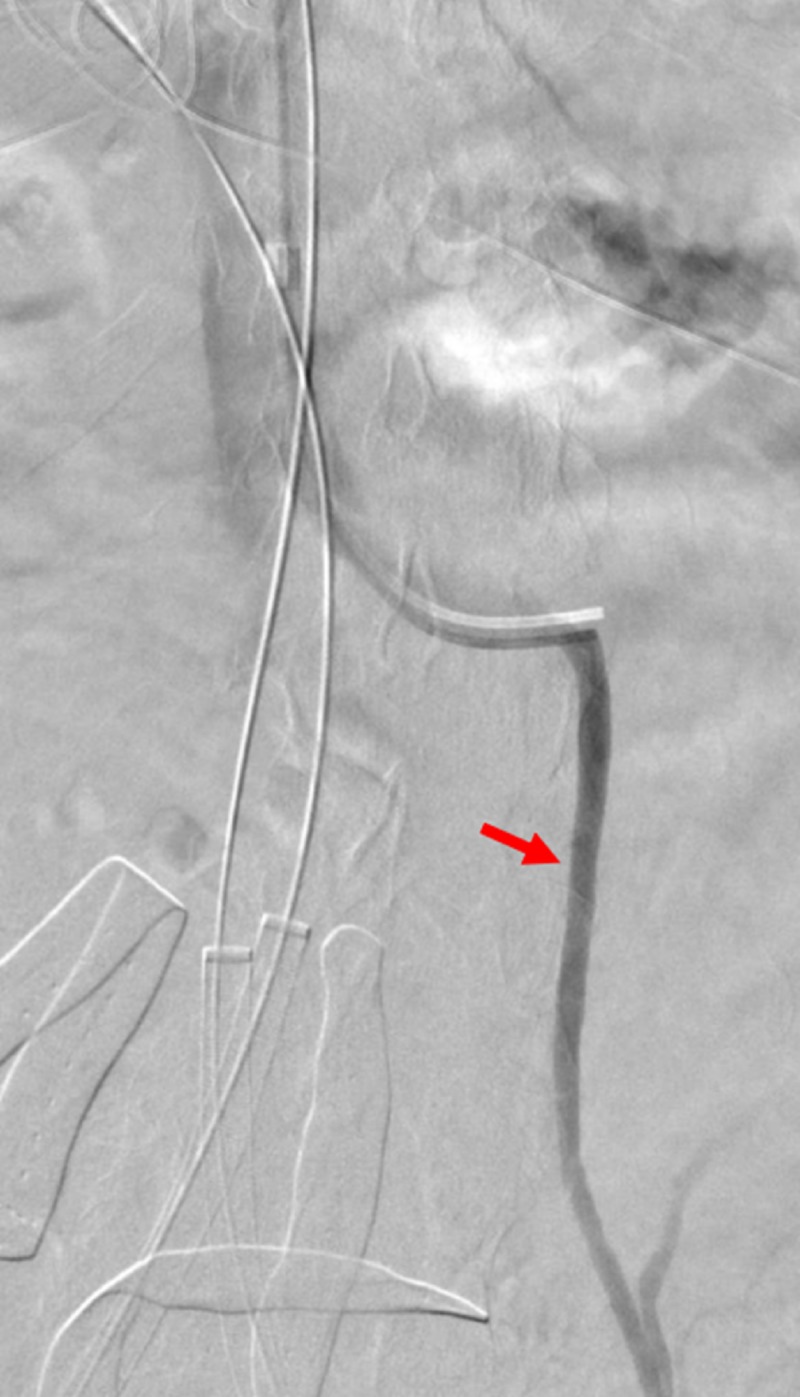
Preparation for stent and IVC placement Bilateral iliac vein sheaths with wires were placed in the preparation for stent placement. A transjugular venous catheter was placed in the left renal vein as a reference for stent and filter placement. The venogram demonstrates reflux into the left gonadal vein (red arrow). IVC: inferior vena cava

**Figure 7:**
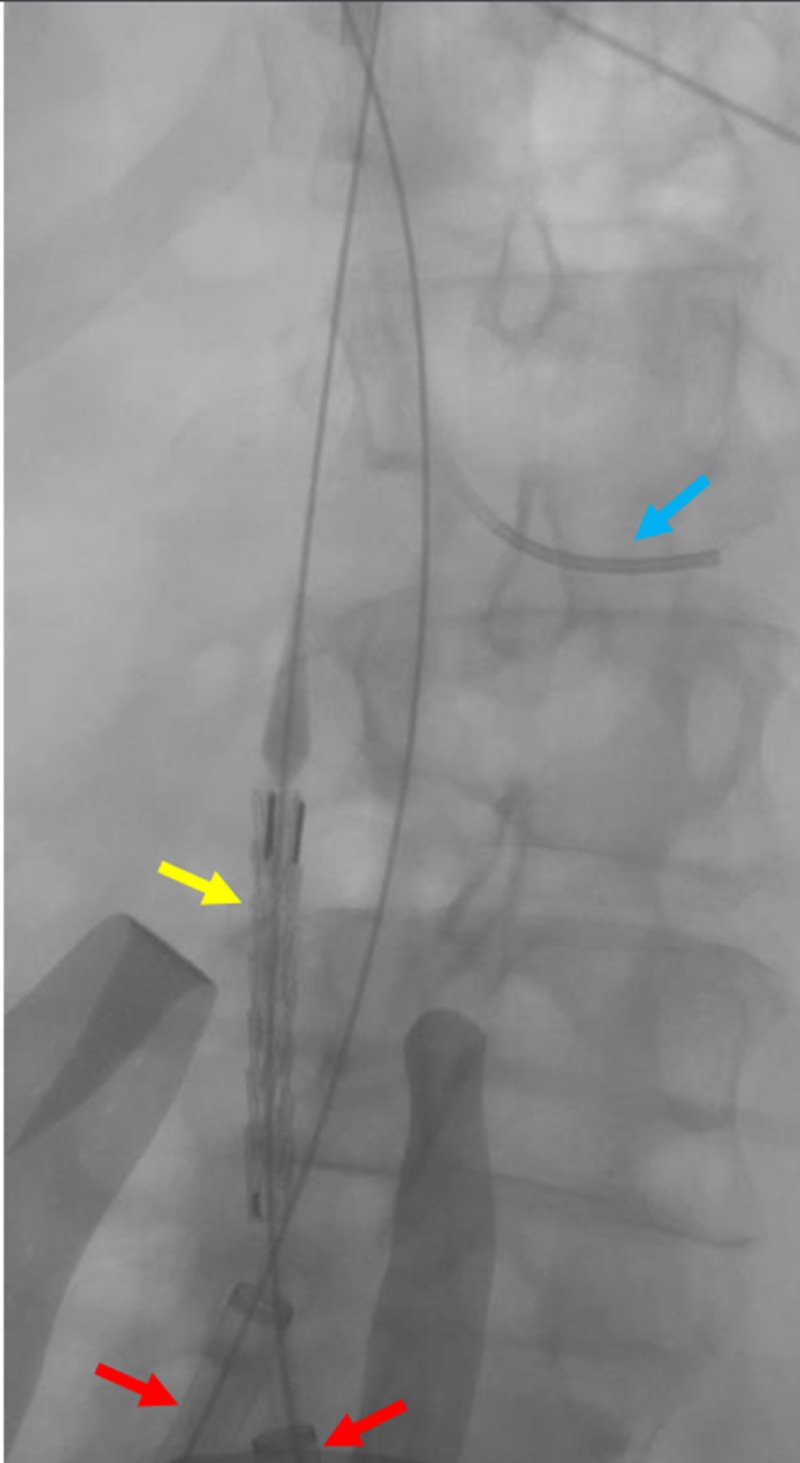
Fluoroscopic view of the IVC before the deployment of a large stent Fluoroscopy is shown to verify the location of the largest (32 mm by 4.5 cm) stent (yellow arrow) within the IVC prior to deployment. The blue arrow indicates a transjugular catheter within the left renal vein as a reference point. The red arrows indicate the bilateral common iliac venous sheaths. IVC: inferior vena cava

**Figure 8:**
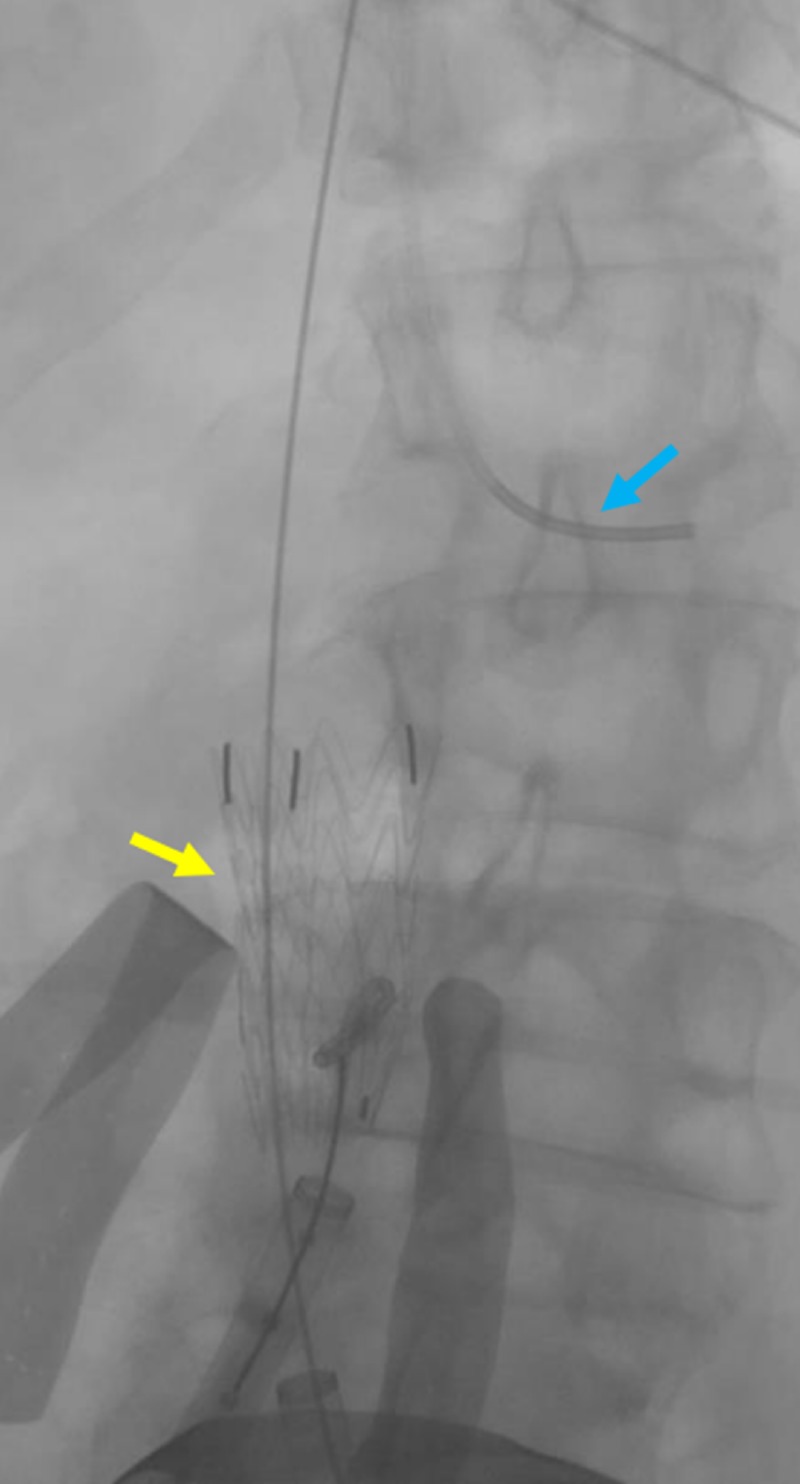
Initial deployment of a large IVC sheath Deployment of the largest sheath (yellow arrow) within the IVC is shown. The blue arrow indicates a transjugular catheter within the left renal vein used as a reference point. IVC: inferior vena cava

Measurements of the IVC from the level of the renal veins to the confluence were obtained. The Gore® excluder stent graft (W.L. Gore & Associates, Flagstaff, AZ) measuring 32 mm by 4.5 cm was uneventfully deployed within the proximal aspect, marginally below the level of the renal veins (Figures 6-8)

With the calculated native vessel size, the Matteo mathematics method was employed to determine the appropriate sized left and right iliac stent graft diameters:

$$1.05(\pi D_{Native Vessel}) = [(2\pi D_{Stent Graft}) - (2 D_{Native Vessel})]$$

$$1.05(\pi D_{Native Vessel} + 2 D_{Native Vessel}) = 2\pi D_{Stent Graft}$$

With the patient’s calculated IVC native vessel diameter of 24.1 mm, we solved for the appropriately sized side-by-side iliac stent diameters as follows:

$$1.05(\pi 24.1 mm + 48.2 mm) = 2\pi D_{Stent Graft}$$

$$1.05 (123.912382952) = 2\pi D_{Stent Graft}$$

$$20.7073316699 = diameter of stent graft$$

**Figure 9:**
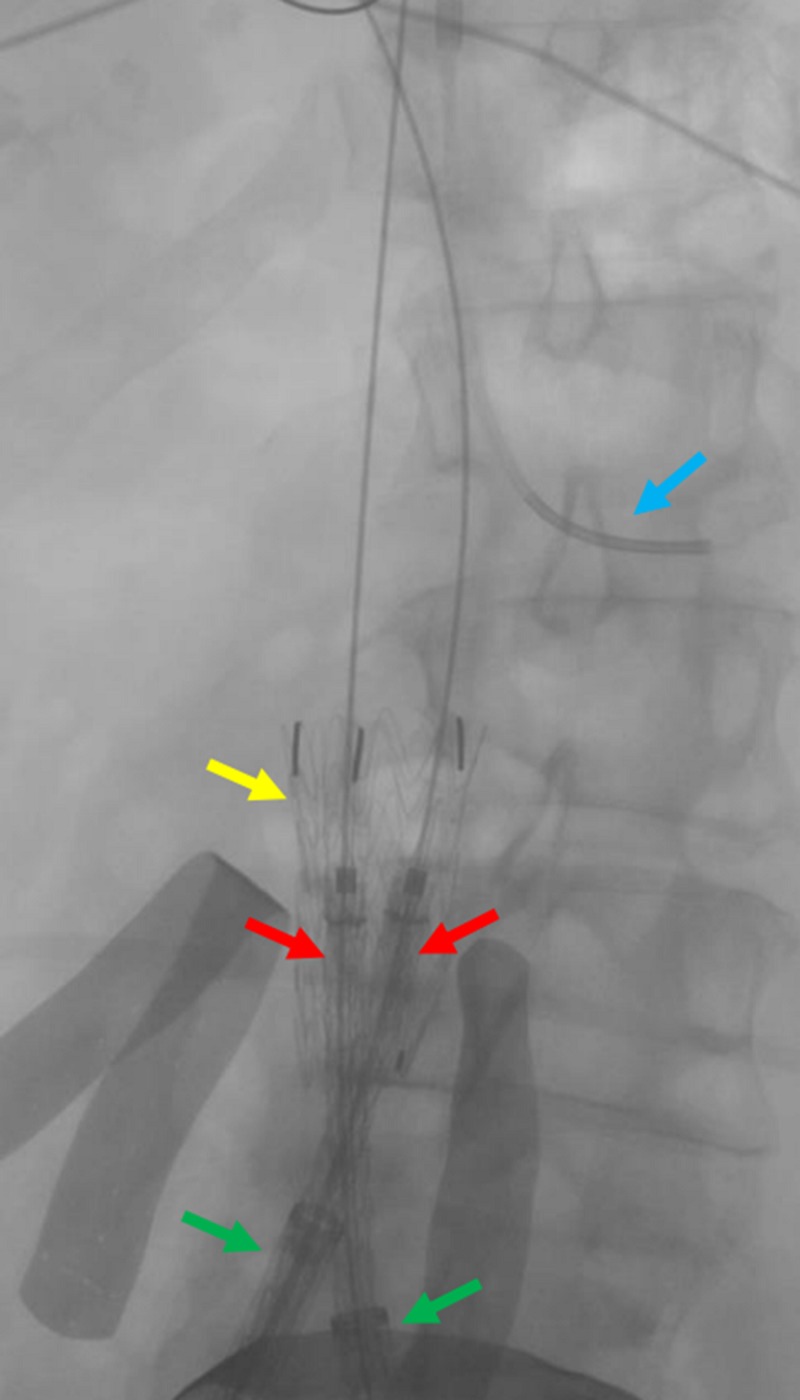
Preparing for the deployment of the common iliac venous stents The fluoroscopic view shows the preparation of the bilateral common iliac venous stents (red arrows) for deployment, overlapping within the IVC stent (yellow arrow). The green arrows indicate the bilateral common iliac venous sheaths. The blue arrow indicates the transjugular venous catheter within the left renal vein. IVC: inferior vena cava

**Figure 10:**
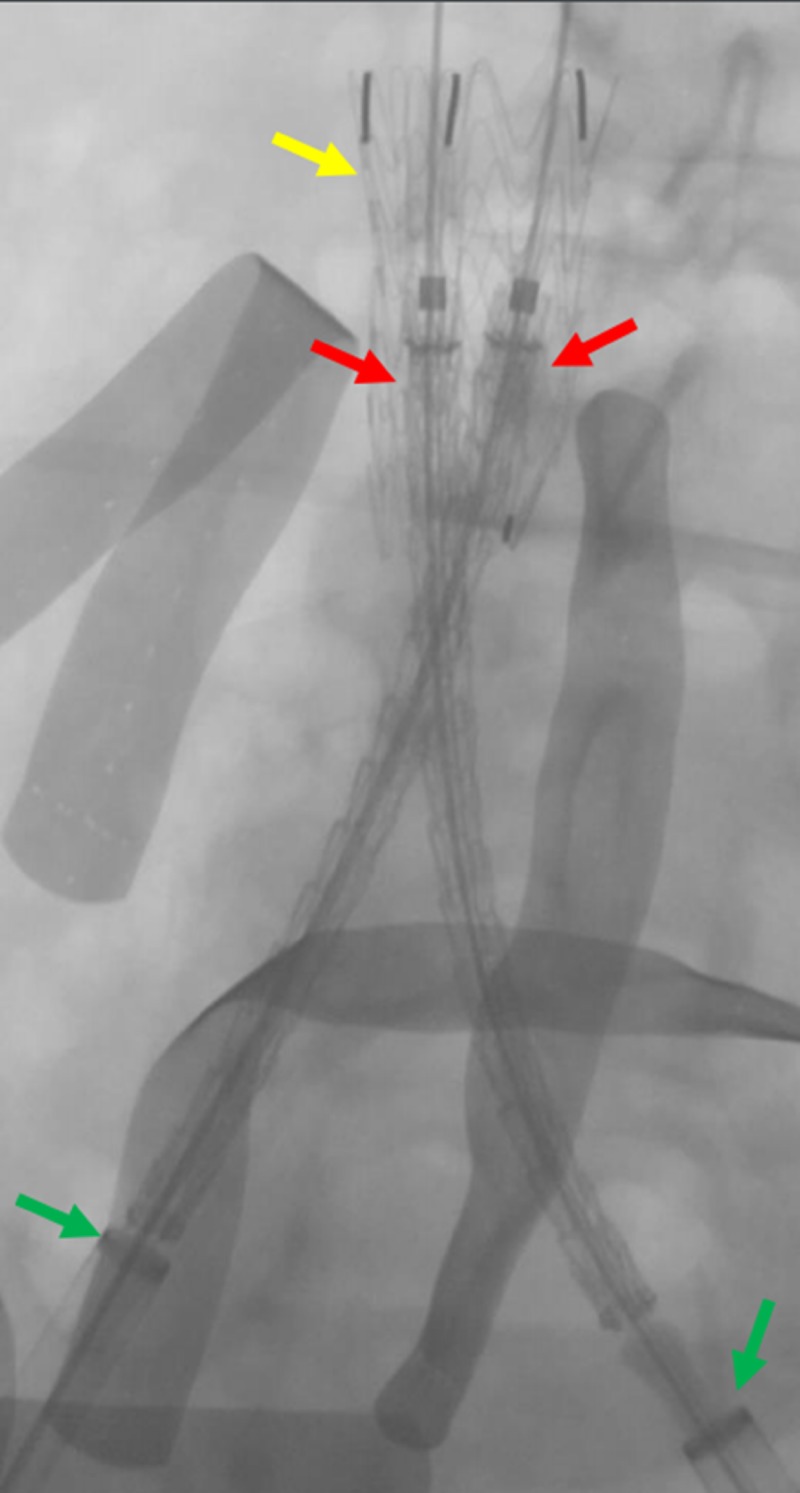
Live deployment of the bilateral common Iliac venous stents Preparing the bilateral common iliac venous stents (red arrows) for deployment within the IVC stent (yellow arrow); green arrows indicate the bilateral common iliac venous sheaths. IVC: inferior vena cava

**Figure 11:**
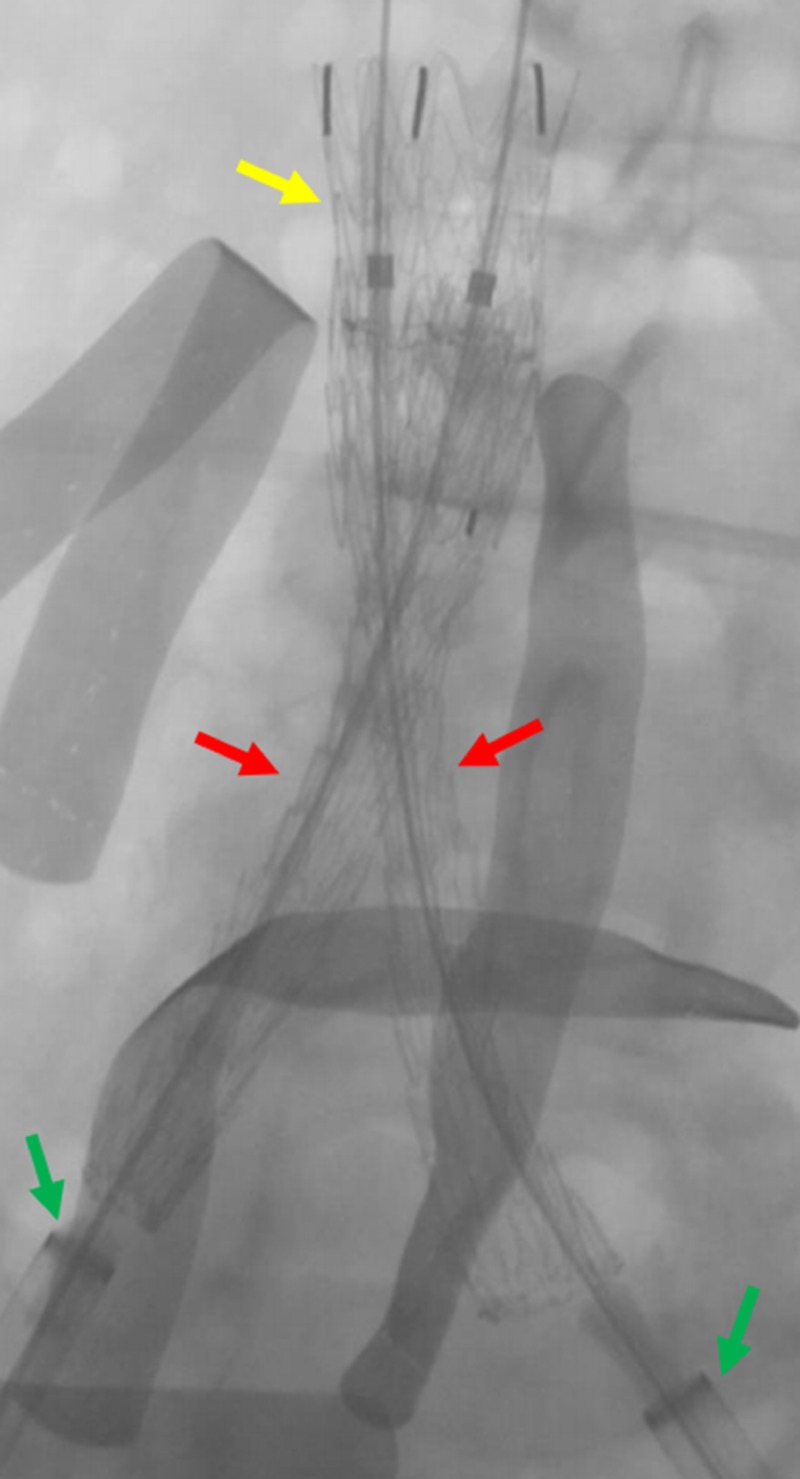
Completed deployment of the bilateral common iliac venous stents The deployment of the bilateral common iliac venous stents (red arrows) within the IVC stent (yellow arrow) is shown. The green arrows indicate the bilateral common iliac venous sheaths. IVC: inferior vena cava

Therefore, two 21 mm by 10 cm diameter Viabahn® stents (W.L. Gore & Associates, Flagstaff, AZ) were chosen to be placed in the right and left iliac veins in a kissing fashion, with the proximal ends terminating within the IVC stent graft (Figures 9-11).

**Figure 12:**
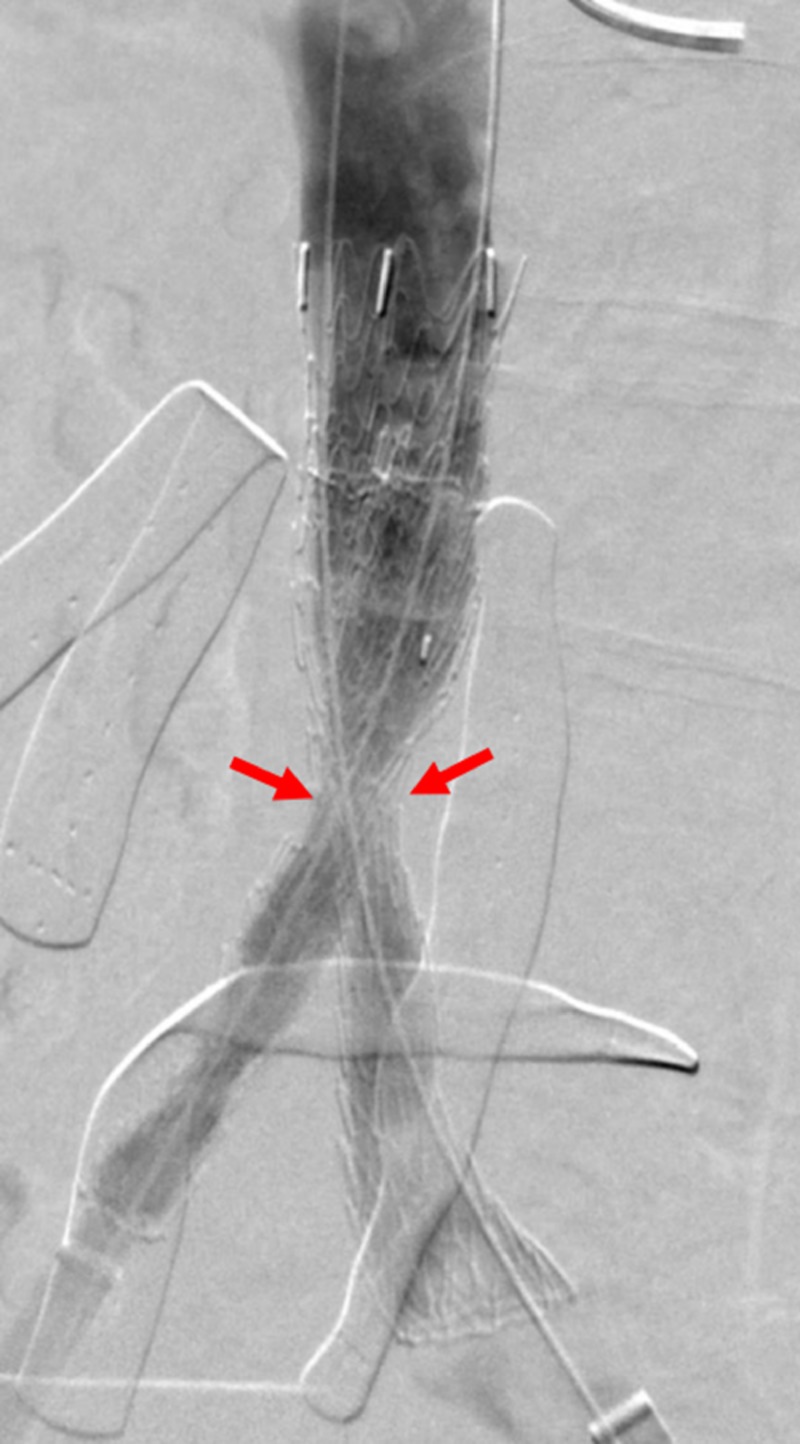
Post stent deployment venogram demonstrating iliocaval stent stenosis The venogram through the iliocaval stents demonstrates stenosis at the iliac confluence (red arrows) due to the nonabsorbable suture placed around the IVC during laparotomy. IVC: inferior vena cava

A subsequent venogram demonstrated stenosis at the iliac vein confluence (Figure 12).

**Figure 13:**
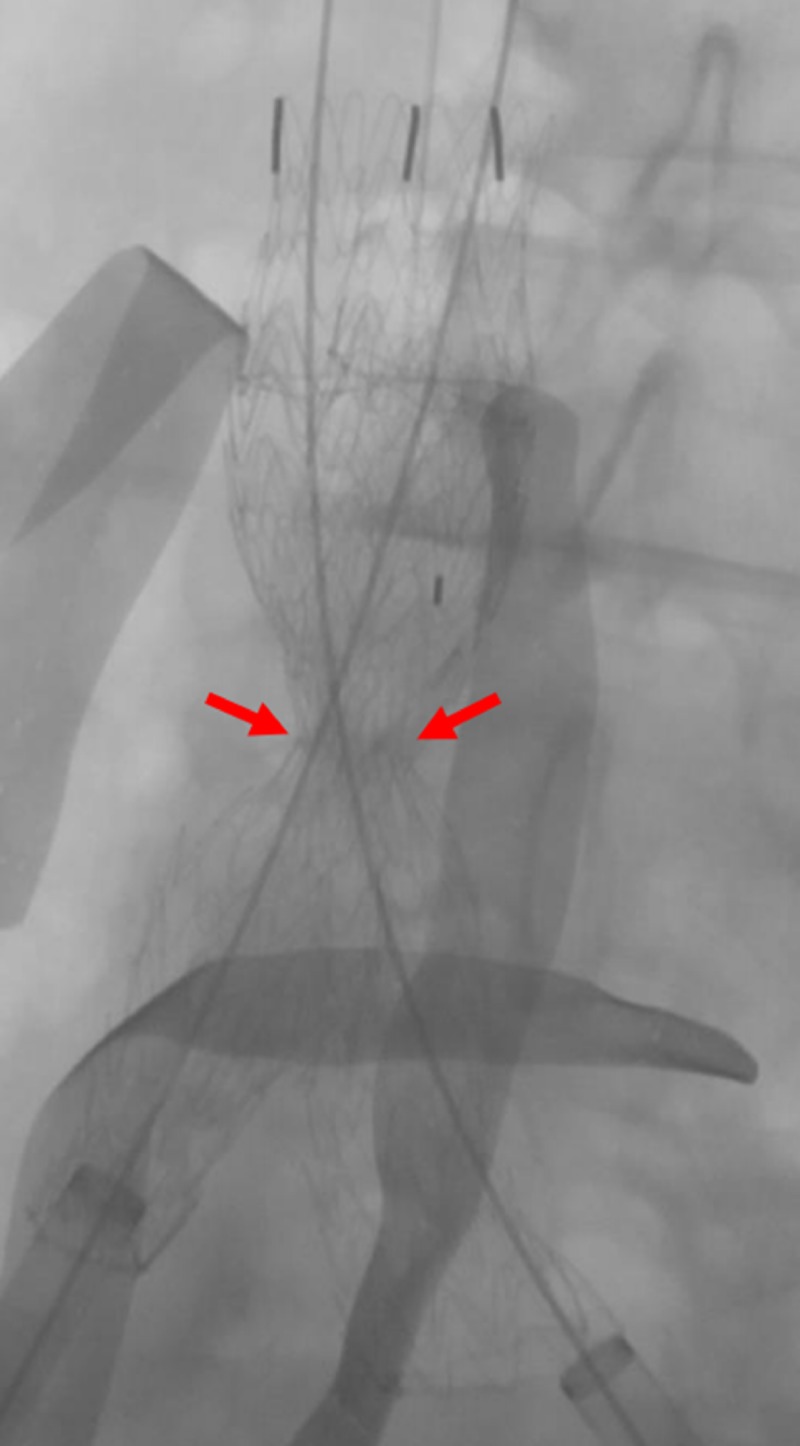
Fluoroscopic View of Iliac Stent Stenosis Before Balloon Angioplasty A fluoroscopic image after transluminal angioplasty demonstrates a persistent waist at the iliac confluence, due to an external nonabsorbable suture placed during laparotomy.

**Figure 14:**
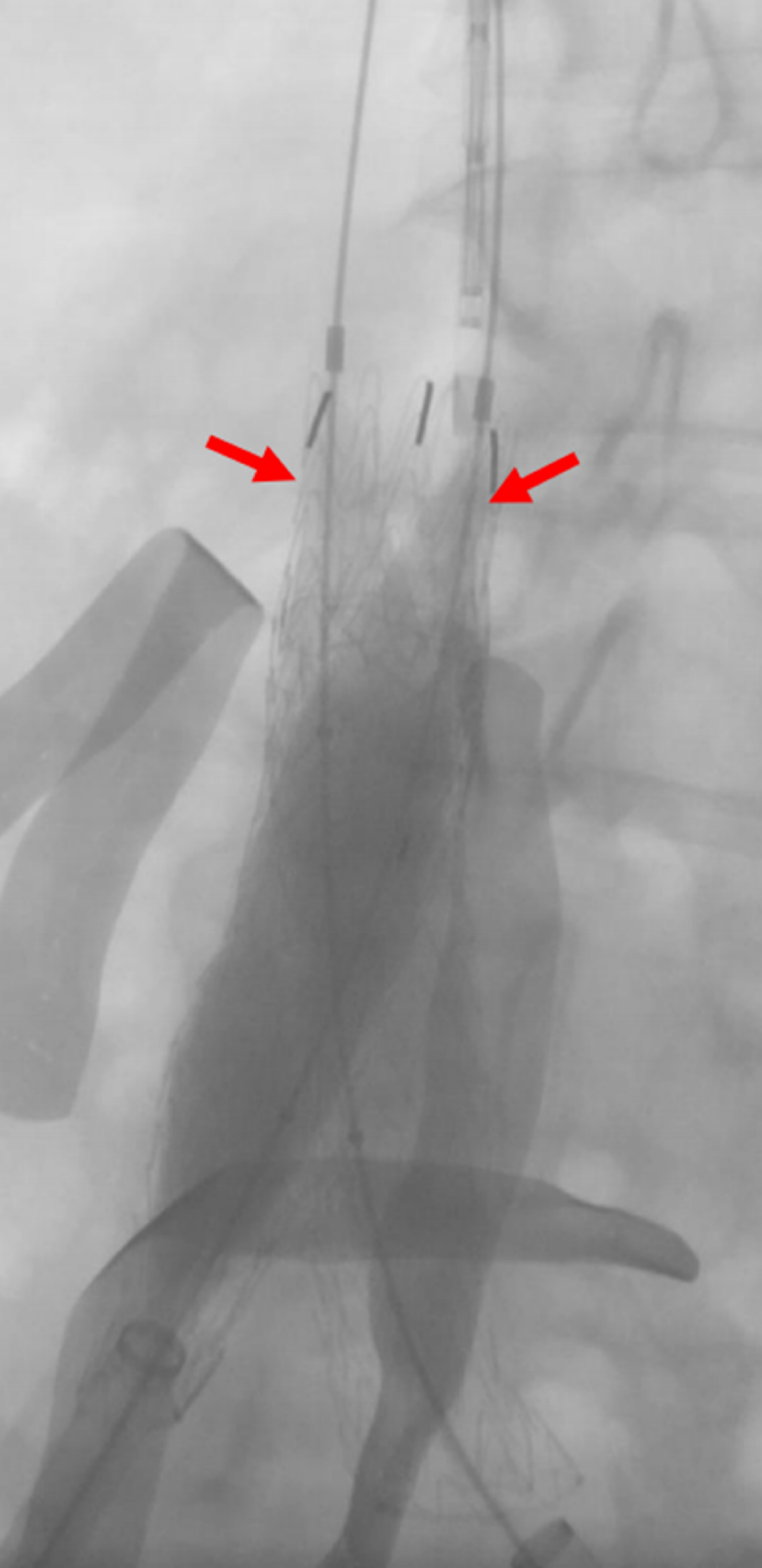
Fluoroscopic view of high-pressure balloons at the level of the iliocaval stenosis A fluoroscopic image demonstrating bilateral simultaneous non-compliant balloons inflated within the iliocaval stents (red arrows) to break the external nonabsorbable suture.

Balloon angioplasty with a 32-mm CODA® (W.L. Gore & Associates, Flagstaff, AZ) balloon was performed throughout the IVC stent graft. A follow-up venogram demonstrated persistent stenosis at the confluence that was subsequently treated with 16-mm Atlas® balloons (C. R. Bard Incorporated, Murray Hill, NJ) inflated simultaneously in a side-by-side fashion (Figures 13-14).

A follow-up venogram demonstrated resolution of the stenosis without endoleaks or contrast extravasation, and the renal veins were shown to be widely patent.

**Figure 15:**
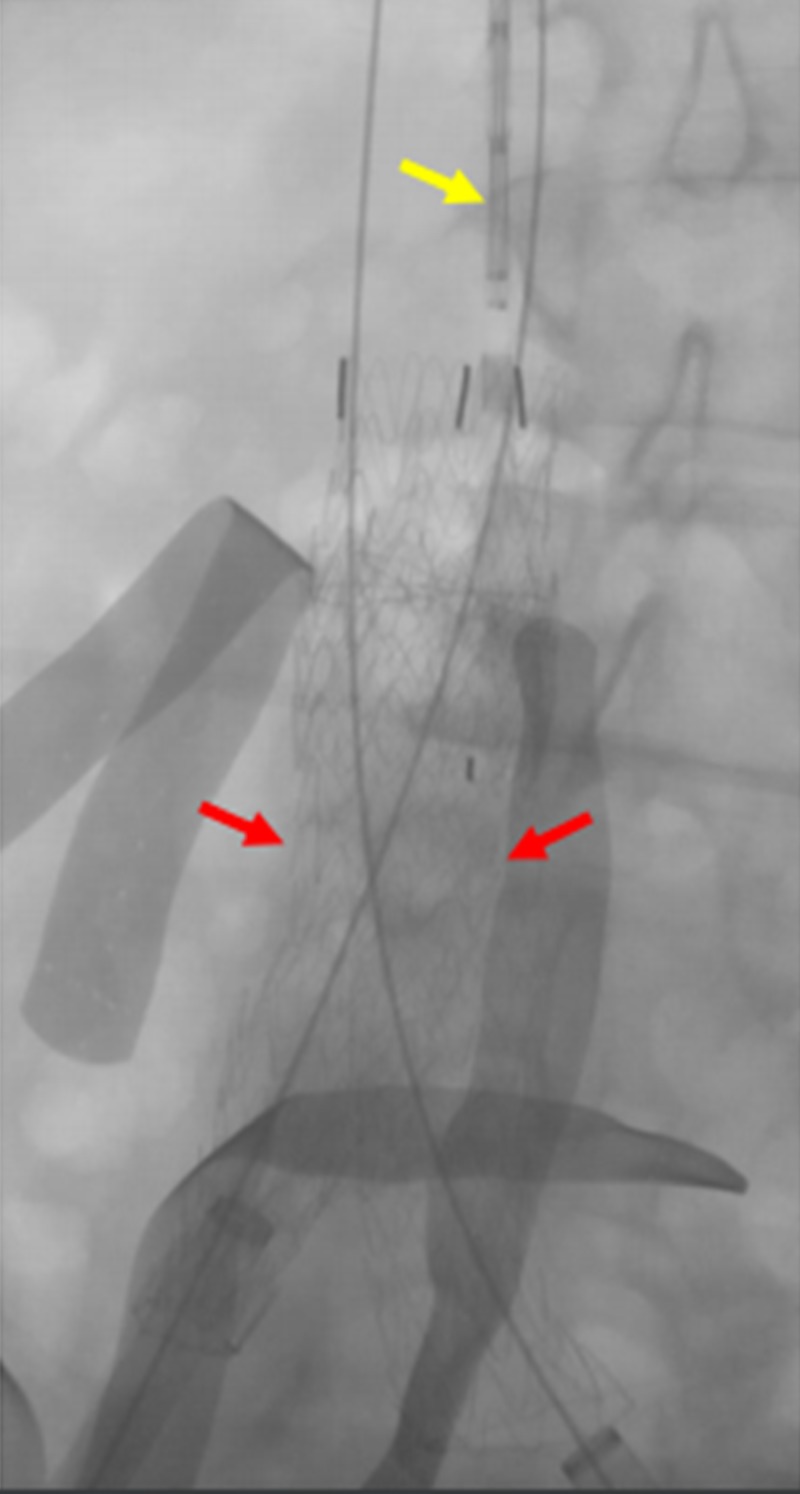
Filter Positioning Before Deployment A fluoroscopic image prior to filter deployment demonstrates the iliocaval stents (red arrows) to be adequately expanded. The IVC filter (yellow arrow) is seen before deployment within the transjugular venous sheath. IVC: inferior vena cava

**Figure 16:**
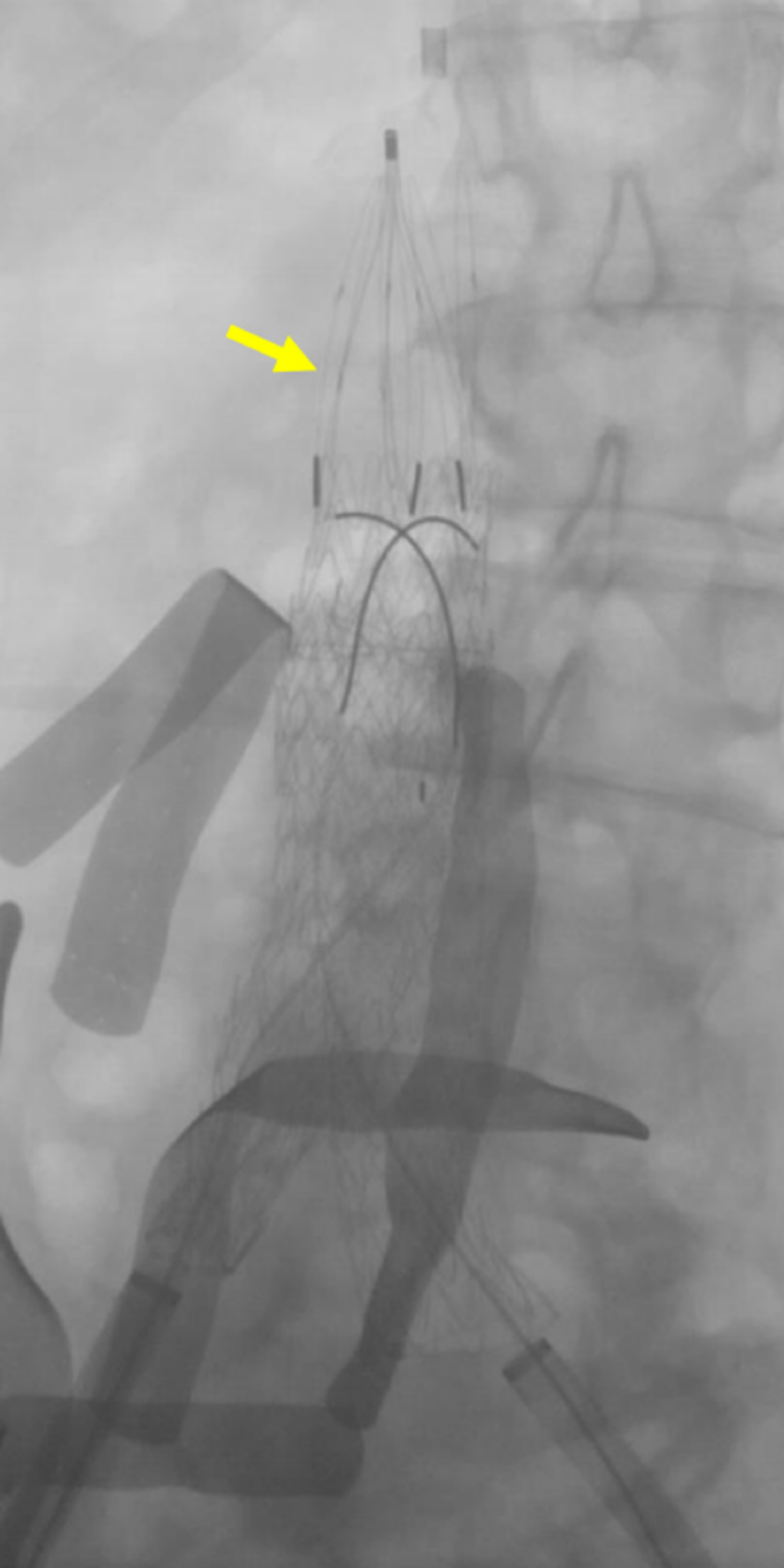
IVC filter deployed above the iliocaval stent The fluoroscopic image demonstrates the permanent IVC filter (yellow arrow) deployed directly above the iliocaval stent. IVC: inferior vena cava

A VenaTech® IVC filter (B. Braun Interventional Systems Incorporated, Bethlehem, Pennsylvania) was then deployed with the apex at the inflow of the renal veins (Figures 15-16).

**Figure 17:**
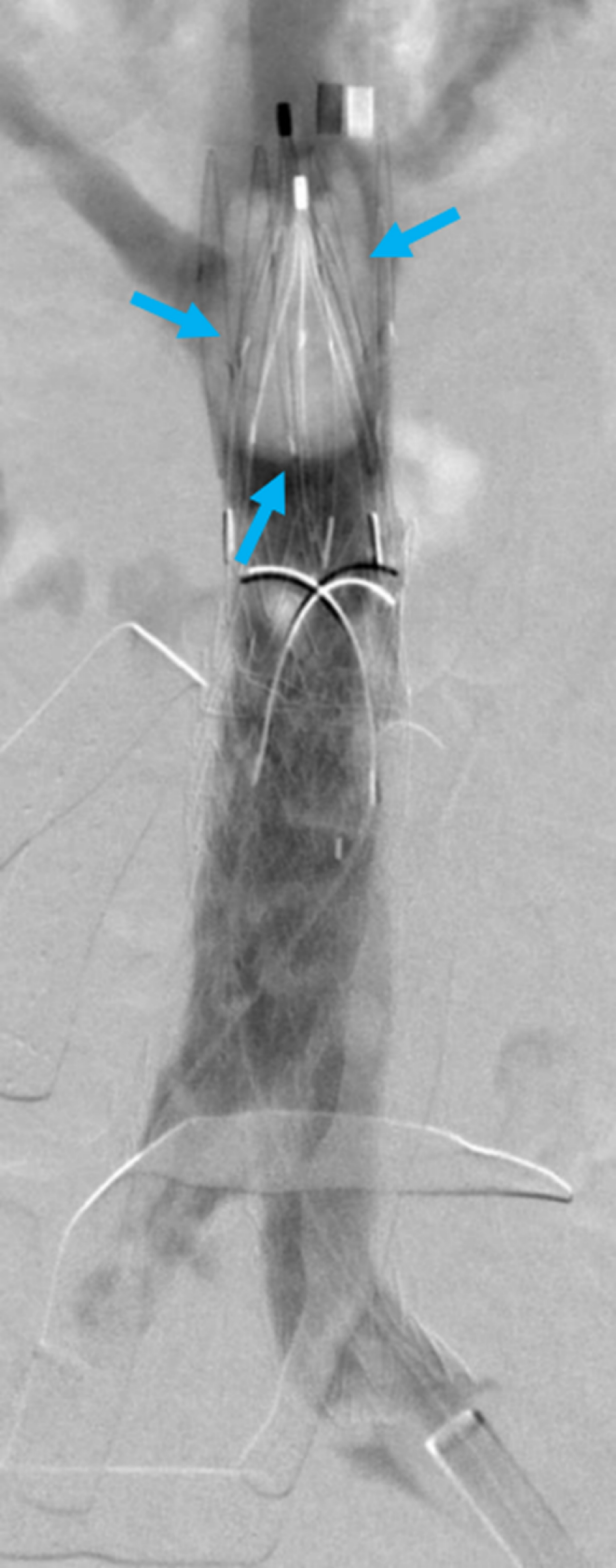
Cavogram demonstrating thrombus formation A cavogram demonstrates the mobile thrombus (blue arrows) lodged within the IVC filter. IVC: inferior vena cava

A follow-up venogram of the iliac vascular sheaths demonstrated an extensive thrombus at the IVC filter, throughout the infrarenal vena cava and visualized common iliac veins (Figure 17).

**Figure 18:**
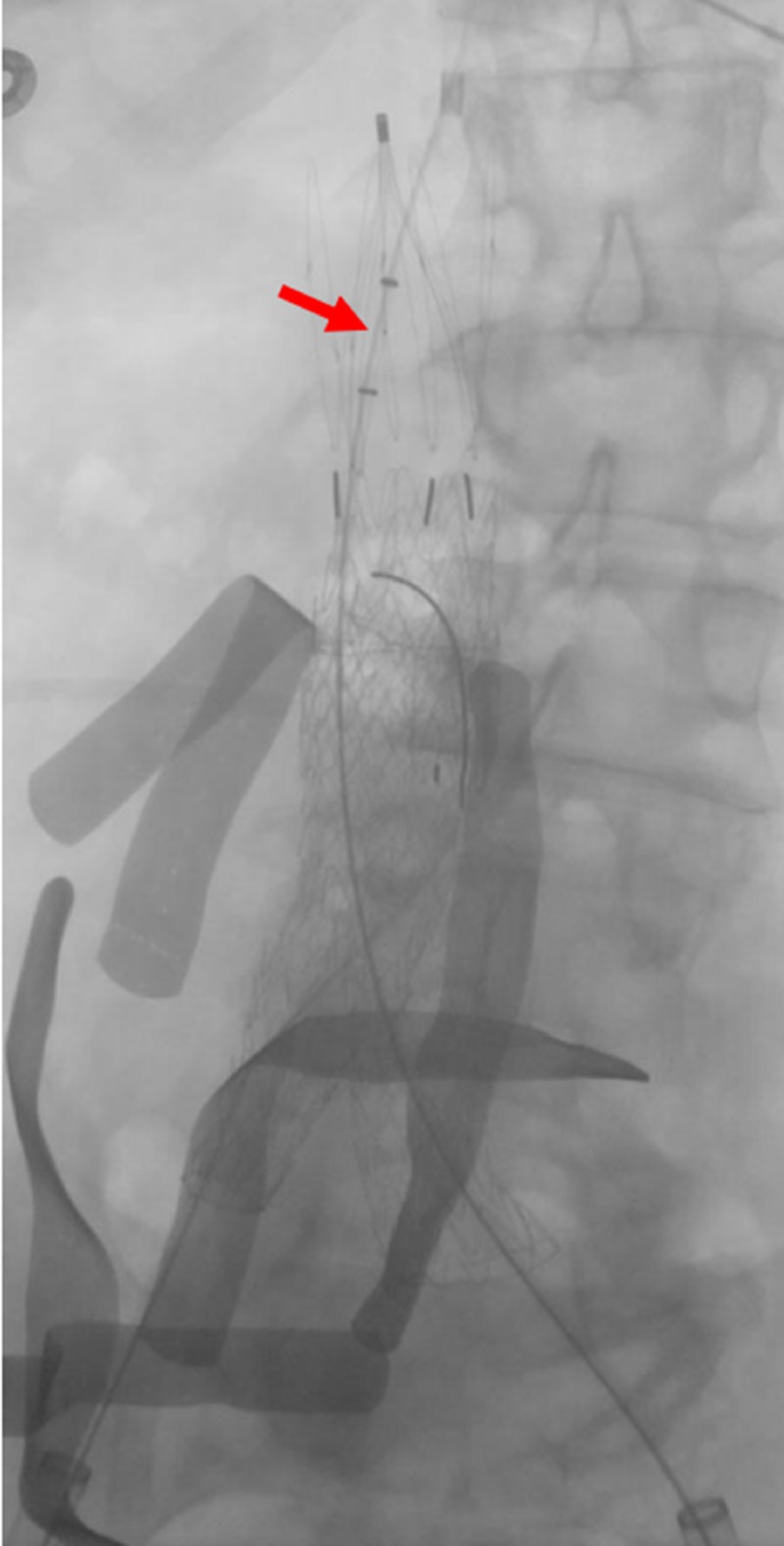
Angiojet® thrombectomy catheter within the IVC filter A fluoroscopic image demonstrates an Angiojet® thrombectomy catheter (red arrow) within the IVC filter. IVC: inferior vena cava

The thrombosis was treated with multiple passes of an eight French Zelante AngioJet® suction thrombectomy catheter (Boston Scientific Corporation, Marlborough, MA; Figure 18).

**Figure 19:**
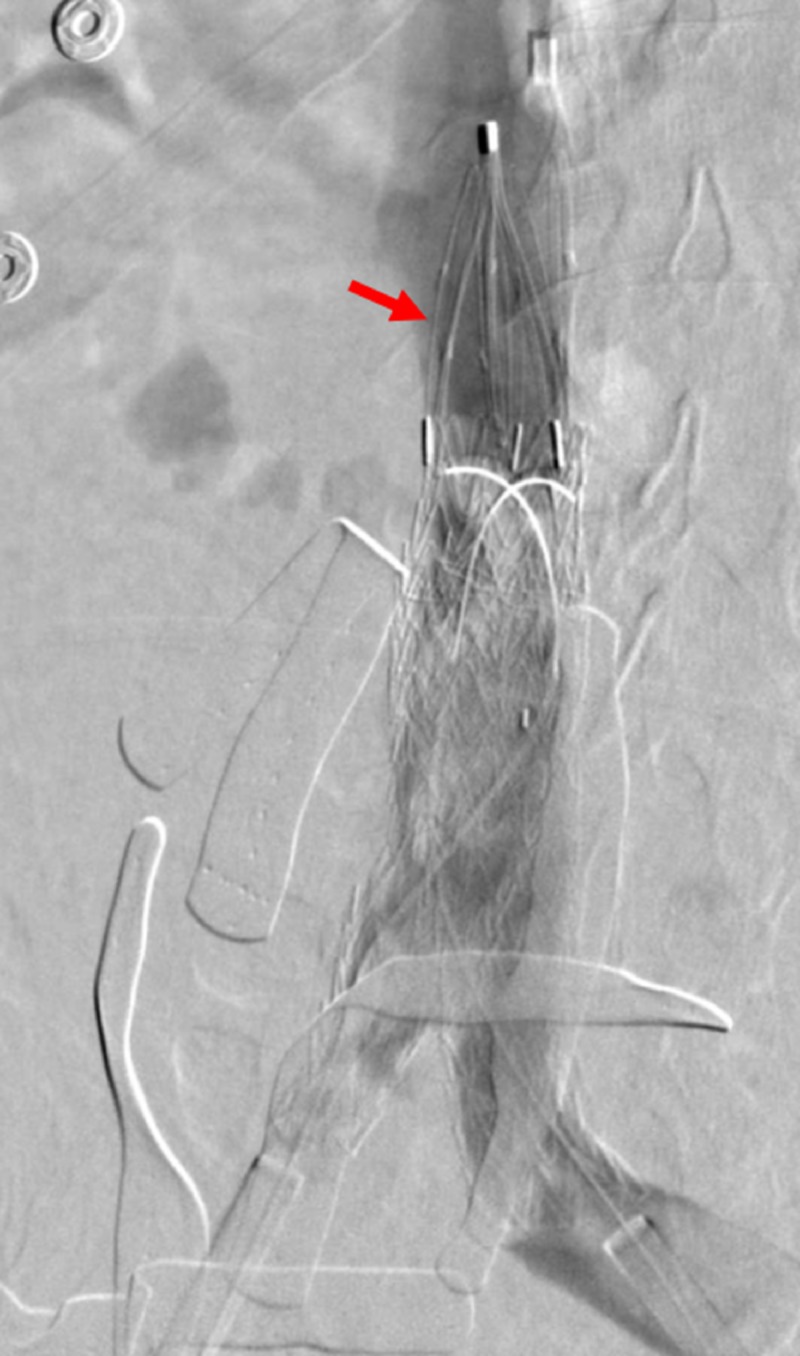
Final cavogram with appropriately placed IVC filter The final cavogram demonstrates no further thrombus within the IVC filter (red arrow). IVC: inferior vena cava

A final venogram demonstrated a significantly decreased thrombus throughout the stent graft and IVC filter (Figure 19).

## Results

**Figure 20:**
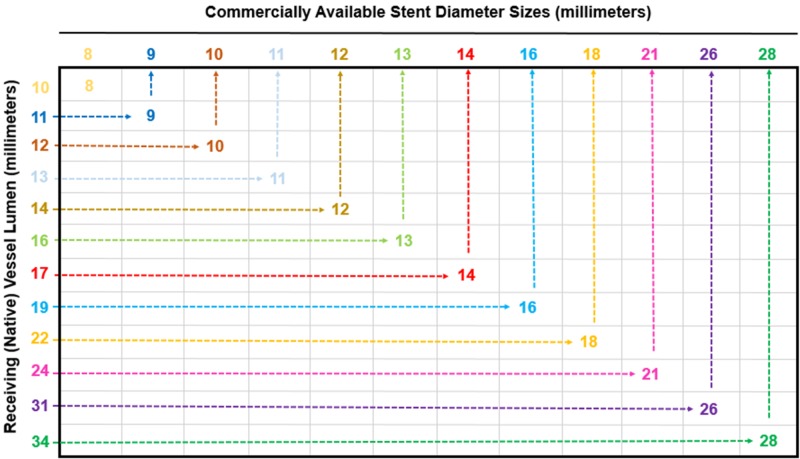
Charted Matteo Mathematics Method Results for Various Native Vessel Lumens and the Corresponding Bilateral Iliac Stent Diameters The table is meant for the purposes of choosing the appropriate iliac stent diameter, when repairing aortic bifurcations or venous confluences, and is based on the Matteo mathematics method. The table only represents the selection of side-by-side iliac stents that are equal in diameter and does not account for kissing stents that are unequal in size. To accurately select the corresponding side-by-side iliac stent diameter for a repair graft, first measure and choose the size of the native vessel lumen (shown on the y axis). Then, follow the native vessel lumen diameter across the corresponding row, until the appropriate iliac stent diameter is met. This is the size that both side-by-side iliac diameters will be in the repair graft.

The Matteo mathematics method was extended from the arterial to the venous system to achieve satisfactory IVC reconstruction with good flow and resolution of contrast extravasation. Especially in the setting of trauma, the predetermined native vessel diameters and corresponding iliac vessel stent sizes would be convenient for the operator to have when placing kissing stent grafts. The following table can be referred to when applying the Matteo mathematics method for venous repair of the iliac veins at the inferior venacaval junction, when bilateral iliac kissing stents of the same diameter are required (Figure 20).

## Discussion

ASVD has conventionally been managed with devices specifically created for the repair of diseased arteries [6]. These repair devices are tailored to the diameter of aortic and iliac vessels, even accommodating diseased bifurcated areas such as the aortoiliac junction [7-8]. However, ASVD repair may be limited by device incapability, including extensive arterial disease that disallows the physical deployment of endovascular devices [5]. Matteo et al. demonstrated a mathematical method to determine side-by-side stent diameter selection based on the native vessel diameter and manufacturer oversize recommendation (five percent in the case of Viabahn® stents) [5].

The case presented demonstrates that the Matteo mathematics method can be adapted to the venous system for stent selection in the setting of venous trauma. Using the aforementioned method, kissing stents are selected as would appropriate for arterial disease, except the native vessel refers to the IVC, where stent diameters are significantly larger than would be required for arterial repair. The diameter size selection in the venous system is notable because larger native vessel diameters require the deployment of larger stents.

For clinical use, the preceding table of the venous native vessel diameters and the corresponding iliac stent sizes has been included. Based on the presentation, whether the vascular etiology is arteriosclerotic repair or trauma, the table can be referred to as a guide when side-by-side stents are needed in iliac vessels of equal diameter. The values reflect even-numbered vessel diameter stent size selections as based on the conventionally available Viabahn® stents; however, the Matteo mathematics method can be calculated and adapted for any iliac stent diameter that is necessary for bifurcation repair. Moreover, the same method can be applied with the use of any other stent, by simply replacing the oversize coefficients recommended for Viahbahn® stents (five percent oversize) with another manufacturer recommendation [9-11].

Though the Matteo mathematics method is applicable to any stent size selection, there is one caveat that interventional radiologists should be aware of; the availability of certain stent diameters may not be produced by any manufacturer. Depending on inventory, bilateral iliac stenting with appropriately sized kissing stents may not be possible for all native vessel sizes. If the calculated stent size necessary for repair is unavailable, upsizing of both iliac stents to the next commercially available size may be required. Furthermore, the operator must consider that the Matteo mathematics method does not account for the repair of iliac vessels with bilateral side-by-side stents of unequal diameter. Though the Matteo mathematics method is limited to bilaterally equal iliac stent selection, the modified Matteo mathematics method may be used to select the kissing stents for unequally sized iliac arteries, caused by ASVD [5].

During the reconstruction of an iatrogenic IVC injury, the Matteo mathematics method was used to calculate the appropriate side-by-side stent sizes needed to repair the venous confluence. The purpose of applying this method is to prevent graft infolding or endoleaks from occurring, preserving the integrity and function of the graft. The Matteo mathematics method should be applied individually to each patient when piecemeal IVC confluence or aortic bifurcation reconstruction is desired using kissing stents.

## Conclusions

The sizing of iliac stent grafts is non-intuitive and requires the calculation of accurate stent diameters. Doing so is critical for the function of stent grafts and for maintaining patency of the vessels. Sizing becomes particularly more complex around areas of bifurcation where kissing stents are needed, whether stents are being placed in the arterial or venous systems. The Matteo mathematics method is a pragmatic way to determine ideal stent size selection and is applicable to IVC confluence reconstruction using kissing stents. Accurate sizing calculations can avoid the potential complications of infolding and endoleaks which can often be due to the sizing discrepancies. This manuscript details an objective and scientific approach for determining stent graft size selection and provides a table which may be referenced in clinical use.
